# Diabetes self-management and its associated factors among patients with diabetes in central Vietnam: A cross-sectional study

**DOI:** 10.1371/journal.pone.0270901

**Published:** 2022-07-08

**Authors:** Van Bang Nguyen, Kim Huong Pham Thi, Thi Xuan Nguyen, Nguyen Tuyen Linh Pham, Van Vy Hau Nguyen, Chi Van Le

**Affiliations:** 1 Center of Endocrinology and Diabetes, Family Hospital, Da Nang, Vietnam; 2 Department of Internal Medicine, Hue University of Medicine and Pharmacy, Hue University, Hue City, Vietnam; Government College University Faisalabad, Pakistan, PAKISTAN

## Abstract

**Objective:**

Diabetes self-management (DSM) enables maintenance of optimal individualized glycemic control for patients with diabetes through comprehensive lifestyle, medication adherence, and self-monitoring glucose level. This study aimed to evaluate DSM and to find associated factors among Vietnamese diabetes patients by using the Vietnamese version of Diabetes Self-Management Instrument (DSMI).

**Methods:**

A cross-sectional study was conducted at a single hospital in the central Vietnam. DSM was assessed using the DSMI. The participant’s socio-demographic and clinical features were obtained through face-to-face interviews and medical records. Multivariate linear regression was used to determine independent factors associated with total DSMI.

**Results:**

The mean total DSM score based on DSMI self-administered questionnaire scores was 88.4 ± 22.1, with a range of 47 to 140. The mean self-integration, self-regulation, interaction with health professionals, self-monitoring blood glucose, and adherence to the prescribed regime were 24.8, 22.3, 21.6, 10.2, and 9.5, respectively. 48.1% of DM patients had good HbA1c control. Sex, educational status, BMI, waist circumference, medical nutrition therapy, and sufficient physical activities were factors independently predictive of DSMI total score.

**Conclusion:**

This study emphasizes that the DSM situation is seen to be average among DM patients with mean DSMI score 88.4 ± 22.1 and sex, educational status, BMI, waist circumference, medical nutrition therapy, and sufficient physical activities were independently predictive factors of DSMI total score. This evidence suggests that there is a need to enhance the effectiveness of DSM education programs among diabetic patients.

## Introduction

Globally, diabetes mellitus (DM) is recognized as one of the four major non-communicable diseases besides cardiovascular disease, cancer, and chronic respiratory diseases. According to the International Diabetes Federation, in 2019 463 million people from 20–79 years of age representing 9.3% of the world’s population are living with DM and its prevalence has been increasing annually [1]. Growing urbanization, sedentary lifestyle, consumption of high caloric food, and stressful lifestyle have led to the abnormal metabolism of carbohydrates, fats, and proteins. Chronic exposure to elevated levels of glucose and lipids triggers various pathways that are responsible to induce impaired insulin secretion from the β-cells of pancreatic islets, insulin resistance in peripheral tissues, decreased glucose utilization in peripheral tissues, and abnormal hepatic glucose production [2, 3]. Diabetes are not only the leading cause of short and long-term health complications, but also one of the top deadly diseases worldwide [4]. While there has been no cure for diabetes, people with diabetes can maintain individualized glycemic control to protect against the development of complications, and to live a healthy life via treatment modalities including lifestyle modification and/or anti-diabetes medications and self-management strategies [5] which are strongly recommended [6]. Notably, the American Diabetes Association (ADA) emphasizes the importance of person-centered care, defined as being respectful of and responsive to the individuals preferences, needs, and values; and ensures that the person with diabetes guides all clinical decisions [7].

Diabetes self-management (DSM) has been defined as how people with diabetes practice self-care. It involves the knowledge, attitude, and behaviors to both maintain personal health and prevent long-term diabetes complications [8]. DSM targets the maintenance of individualized goals for glycemic control through comprehensive lifestyle behaviors including dietary management, physical activity, and weight management, optimizing medication taking behaviors, and self-monitoring of glucose [9]. Several studies have revealed that improving DSM supports achieving improved health outcomes and reducing the incidence of complications [10–12]. According to McDowell and colleagues, DSM may be as efficacious as diabetes medications in maintaining glucose levels, especially in those with newly diagnosed DM [13]. Studies have indicated a high rates of suboptimal DSM skills in people with DM despite strong evidence of a positive link between DSM and glycemic control [14, 15]. Difficulties in coping with diabetes, self-monitoring skill deficits, and lifestyle challenges are among the many barriers in promoting DSM to people living with diabetes [16–18].

Because DSM and patient-centered care are cornerstones of successful diabetes care, over 30 validated instruments have been developed to investigate its features, prevalence, and related factors which impact DSM. These tools include the Adherence and Self-Management Monitoring Tool (ASMMT) [19], Diabetes Care Profile (DCP) [20], Diabetes Health Promotion Self-Care Scale (DHPSC) [21], Diabetes Self-Care Ability Questionnaire (DSCAQ) [22], and the Diabetes Self-management Assessment Report Tool (D-SMART) [23]. They majority of these surveys allow evaluation of multiple dimensions of core diabetes treatment such as diet, physical activity, medication, self-monitoring of blood glucose, foot care, interactions with a physician, and management of hypoglycemia [24].

The Diabetes Self-Management Instrument (DSMI) is one of the validated tools for assessing DSM. The DSMI was developed in 2008 by Lin and his colleagues to evaluate the self-management behaviors of patients with diabetes [25]. It is a multidimensional instrument, which includes 35 items (DSMI-35) divided into 5 subscales: self-integration (10 items), self-regulation (9 items), interaction with health care providers (HCPs) and significant others (9 items), self-monitoring of blood glucose (SMBG) (4 items), and adherence to the recommended regimen (3 items). The DSMI has generated evidence that DSM is an active and flexible process in which people with DM develop strategies for achieving their desired goals by regulating their own actions, collaborating with HCPs and several additional factors including psychosocial domains. The DSMI has been translated and validated as a reliable instrument with high internal consistency in different nations including Vietnam [26–29]. Recently, the DSMI was shortened to 20 items for practical purposes [30]. However, the Vietnamese version of the original DSMI remains a 35 item instrument with Cronbach’s alpha = 0.92 [28].

In Vietnam, DM is rising at a rate of about 6.23% every year, and it has become a serious health and economic burden for Vietnamese society [31]. Therefore, evaluation of DSM has been intergrated into treatment, patient education, and patient-centered care policy. To the best of our knowledge, there has been a dearth of previous studies of DSM and its related factors in patients with diabetes in Vietnam, except for the study by Dao Tran and her colleagues asessing the test-retest reliability and criterion validity of the Vietnamese version of DSMI [28]. In this study, we aimed to evaluate the current state of DSM and to identify factors associated among Vietnamese DM patients by using the Vietnamese version of DSMI.

## Methods

### Study design and sampling

From March 2021 to May 2021, we conducted a cross-sectional study among outpatients at the Center of Endocrinology and Diabetes, Da Nang Family Hospital, Da Nang, Vietnam. Patients with DM were invited to enroll in this study if they met the following inclusion criteria: 1) A diabetes diagnosis per the ADA 2021 criteria for diagnosis made at least 3 months prior to study entry [32]; 2) voluntarily willing to participate 3) capable of understanding and responding to the questionnaire; 4) currently not be experiencing an acute and serious medical illness. To evaluate the mean DSMI score in participants, the sample size was calculated by applying a formula for an estimated single mean with specified precision. With a 95% confidence interval, a margin of error of 3, and the anticipated standard deviation of DSM among patients with diabetes at 16.89 (following the previous study [33]), the sample size for this study was calculated to be 125 participants [34]. With an anticipated 10% for a refusal rate, the final sample size for the study was 137 participants.

Potentially eligible participants were identified during clinic visits using convenience sampling at the Center of Endocrinology and Diabetes, Da Nang Family Hospital. Nurses were trained in administration of the DSMI and participants completed the survey during face-to-face visits. A structured questionnaire and the hospital electronic medical records system were used to collect sociodemographic and clinical information.

### Ethical approval

This study was conducted in accordance with the Declaration of Helsinki. The Institutional Review Board of the Danang Family Hospital (number: 71.01–30303) approved the study protocol prior to initiation of study activities. Each participant was informed of the purposes of this study in detail via an information sheet and provided informed consent form if they agreed to join the study. Participants were free to withdraw at any time, without giving any reason for doing so and without affecting their present or future medical treatment. All participant information was kept confidential and used only for study purposes.

### Data measurements

#### Socio-demographic information

Socio-demographic variables consisted of age (continuous variables, age groups: > 60 years old and ≤ 60 years old), sex (categorical variables: male and female), occupation (categorical variables: retirement, officers, self-employed, and others), family status (categorical variables: single, married, and widow/divorced), living area (categorical variables: urban areas and others), and academic background (categorical variables: illiterate, primary school (grade 1–5), secondary/high school (grade 6–12), and vocational/college).

#### Clinical features

Clinical information consisted of the types of DM (categorical variables: type 1 and type 2), duration of living with DM (continuous variable, years, grouped: ≤ 5 years and > 5 years), blood pressure (continuous variable, mmHg), body mass index (continuous variable; kg/m^2^, grouped following Asia-Pacific body mass index classifications [35]: Low/normal and Overweight/Obese), waist circumference (continuous variable, cm, grouped: android obese or not), antihyperglycemic drugs (categorical variable: oral antidiabetic drugs (OAD), OAD plus insulin, and insulin only), medical nutrition therapy (MNT) (categorical variables: yes and no), sufficient physical activity (categorical variables: yes and no), fasting blood glucose level (continuous variable, mmol/l), lipid profile (continuous variable, mmol/l), and HbA1c (continuous variable, %).

#### The Diabetes Self-Management Instrument (DSMI)

The DSMI consists of 35 items used to measure self-care of the person with DM in 5 domains: self-integration, having 10 items related to the “ability to integrate diabetes care into day-to-day activities like appropriate diet, physical activity and control of weight”; self-regulation, having 9 items related to the “self-regulation of the behavior via monitoring of physical symptoms about diabetes”; interaction with HCPs, having 9 items related to the need of HCPs in diabetes care”; SMBG, having 4 items related to the “monitoring of blood glucose in order to accommodate self-care behaviors”; and medication adherence, having 3 items related to “diabetes patients’ adherence to medication and clinic visit” [25]. Each item was rated on a 5-point Likert scale (1, considering as never; 2, considering as rarely; 3, considering as sometimes; 4, considering as usually; and 5 considering as always). Therefore, the total possible DSMI score range was 35 to 175. The ranges of the scores for self-integration, self-regulation, interaction with HCPs, SMBG, and medication adherence were 10 to 50, 9 to 45, 9 to 45, 4 to 20, and 3 to 15, respectively. The reliability of the original DSMI achieved a Cronbach’s alpha coefficient of .94 and a test-retest correlation of 0.73 [25]. In this study, conducted in 2021, the Vietnamese version of the DSMI (V-DSMI) was translated and validated in Vietnamese patients by Dao-Tran and her colleagues with Cronbach’s alpha coefficient: 0.81–0.95 for each domain and 0.91 for the overall. V-DSMI showed the acceptability and appropriateness for Vietnamese diabetes patients [28].

### Data analysis

To perform all data analysis, SPSS software version 20.0 for Windows was used. Baseline patient characteristics were summarized using means and standard deviations for continuous variables, and counts and percentages for categorical variables. Independent-samples T-test and ANOVA were used to compare the total score of DSMI between two or more independent groups. The Mann-Whitney U test and Kruskal-Wallis test were used if data could not be assumed to be normally distributed. A p-value < 0.05 was considered statistical significance. To identify the factors that predicted DSM behavior in participants, multivariate linear regression was employed. A backward selection strategy, which started with all factors in the model, was used to iteratively remove the least contributive predictors and to choose the best and final model for data analysis with a conventional p-value threshold of 0.05.

## Results

A total of 137 participants consented to join the study among whom 108 (78.3%) completed the V-DSMI questionnaire. Among non-completers, 20 withdrew because of the COVID-19 pandemic and 10 lacked results of study blood tests.

The socio-demographics of the participants are shown in Table 1. They were all Vietnamese. The sample was 50% male and the the mean age was 56.6 years (SD, 11.5). The majority had an education background of secondary school or above (74.1%) and lived with relatives (98.1%) in urban areas (78.7%). Fully 95.4% had a diagnosis of type 2 diabetes and 49.1% had been living with DM for more than 5 years. Relative to lifestyle behaviors, 38.0% self-reported following a medical nutrition therapy plan and 68.5% participating in sufficient physical activity.

**Table 1:** Socio-demographic characteristics of DM participants.

Characteristics	Total (n = 108)		Characteristics	Total (n = 108)	
	n	%		n	%
**Sex**			**BMI**		
Female	54	50.0	Low/normal	36	33.3
Male	54	50.0	Overweight/Obese	72	67.7
**Age, (year), [mean** ± **SD]**	56.6 ± 11.5		**Hypertension**		
<60	62	57.4	Yes	30	27.8
≥60	46	42.6	No	78	72.2
**Occupation**			**Current treatment regime**		
Retirement	25	23.1	OAD	74	68.5
Officers	16	14.8	OAD+ insulin	22	20.4
Self-employed	20	18.5	Insulin alone	12	11.1
Others	47	43.5			
**Living area**			**Android obesity**		
Urban areas	85	78.7	Yes	72	33.3
Others	23	21.3	No	36	66.7
**Educational status**			**Medical nutrition therapy**		
Literate	12	11.1	Yes	41	38.0
Primary school	16	14.8	No	67	62.0
Secondary/high school	48	44.4			
Vocational/college	32	29.7			
**Marital status**			**Sufficient physical activity**		
Single	4	3.7	Yes	74	68.5
Married	98	90.7	No	34	31.5
Widow/Divorced	6	5.6			
**Living arrangement**			**Dyslipidemia**		
Living alone	2	1.9	Yes	94	87.0
Living with relatives	106	98.1	No	14	13
**Duration of diabetes (years)**			**HbA1c (%), [mean** ± **SD]**	7.6 ± 1.8	
≤5	55	50.9	<7	52	48.1
>5	53	49.1	≥7	56	52.9
**Type of diabetets**			**Fasting plasma glucose (mmol/l), [mean** ± **SD]**	8.0 ± 2.8	
Type 1	5	4.6	Good	53	49.1
Type 2	103	95.4	Not good	55	50.9

SD, Standard Deviation; OAD, Oral Antidiabetic Drugs

The average HbA1c was 7.6% (SD, 1.8) and fasting blood glucose levels were 8.0 mmol/l (SD, 2.8). According to ADA criteria 48.1% had good HbA1c control (HbA1c < 7%) and 49.1% good fasting plasma glucose control (FPG: 4.4–7.2 mmol/l). The prevalence of overweight and obesity, android obesity and dyslipidemia were 67.7%, 33.3%. and 87.0%, respectively.

The DSM and its internal consistency are shown in Table 2. The mean total DSM score based on the V-DSMI self-administered questionnaire was 88.4 ± 22.1, with a range of 47 to 140. The score for mean self-integration was 24.8, for self-regulation 22.3, interaction with health professionals 21.6, self-monitoring of blood glucose 10.2, and adherence to the recommended regimen 9.5.

**Table 2:** Diabetes self-management scores and internal consistency.

Subscales	Number of items	X ± SD	Range	
			min	max
Self-integration	10	24.8 ± 6.9	12	40
Self-regulation	9	22.3 ± 5.9	11	36
Interaction with health professionals	9	21.6 ± 7.6	9	36
Self-monitoring of blood glucose	4	10.2 ± 2.6	5	16
Adherence to recommended regimen	3	9.5 ± 1.6	4	12
Total	35	88.4 ± 22.1	47	140

Table 3 shows a comparison of the mean total DSMI scores across groups of participants by socio-demographic characteristics. Retired participants had higher mean DSM scores than those who were officers, self-employed, and others (p = 0.035). Those with a higher educational status had a higher mean total DSMI score (p = 0.0001). The mean total DSMI scores in subgroups by other socio-demographic features were not significantly different (p > 0.05).

**Table 3:** The mean total DSMI scores across groups by socio-demographic characteristics.

Characteristics	Total (n = 108)	X ± SD	p-value
	n		
**Sex**			
Female	54	89.76 ± 19.48	0.465
Male	54	87.09 ± 24.54	
**Age, (year),**			
<60	62	88.37 ± 22.76	0.98
≥60	46	88.50 ± 21.41	
**Occupation**			
Retired	25	97.80 ± 21.63	0.035
Officers	16	93.69 ± 25.82	
Self-employed	20	84.00 ± 22.04	
Others	47	83.53 ± 19.56	
**Living area**			
Urban areas	85	89.89 ± 21.63	0.29
Others	23	83.00 ± 23.42	
**Educational status**			
Literate	12	69.00 ± 15.33	0.0001
Primary school	16	81.93 ± 20.30	
Secondary/high school	48	87.54 ± 21.64	
Vocational/college	32	100.28 ± 19.43	
**Marital status**			
Single	4	92.50 ± 14.73	0.422
Married	98	89.02 ± 22.07	
Widow/Divorced	6	76.00 ± 25.58	
**Living arrangement**			
Living alone	2	112.50 ± 7.77	0.068
Living with relatives	106	87.97 ± 22.04	

Table 4 gives information on the mean DSMI total score in accordance with clinical characteristics. The mean DSMI total score was found to be significantly higher in participants with medical nutrition therapy (p = 0.002), sufficient physical activities (p = 0.0001), and HbA1c < 7% (p = 0.011).

**Table 4:** Clinical characteristics and the mean total DSMI scores by clinical characteristics.

Characteristics	Total (n = 108)	X ± SD	p-value
	n		
**Type of diabetets**			
Type 1	5	95.40 ± 30.84	0.392
Type 2	103	88.08 ± 21.73	
**BMI**			
Low/normal	36	92.61 ± 20.87	0.117
Overweight/Obese	72	86.33 ± 22.52	
**Duration of diabetes (years)**			
≤5	55	86.72 ± 23.14	0.431
>5	53	90.19 ± 21.02	
**Current treatment regime**			
OAD	74	89.08 ± 23.07	0.814
OAD+ insulin	22	86.91 ± 15.77	
Insulin only	12	87.17 ± 27.09	
**Hypertension**			
Yes	30	89.33 ± 20.14	0.773
No	78	88.08 ± 22.92	
**Android obesity**			
Yes	72	86.31 ± 21.49	0.281
No	36	92.67 ± 22.98	
**Medical nutrition therapy**			
Yes	41	96.90 ± 17.29	0.002
No	67	83.23 ± 23.20	
**Sufficient physical activity**			
Yes	74	95.70 ± 19.25	0.0001
No	34	72.59 ± 19.62	
**HbA1c (%)**			
<7	52	93.98 ± 20.58	0.011
≥7	56	83.26 ± 22.36	
**Fasting plasma glucose control**			
Good	53	85.15 ± 22.39	0.185
Not good	55	91.58 ± 21.52	
**Dyslipidemia**			
Yes	94	88.86 ± 20.81	0.661
No	14	85.50 ± 30.13	

SD, Standard Deviation; OAD, Oral Antidiabetic Drugs

The independently predictive factors of the DSMI total score resulted from multivariate linear regression are shown in Table 5. We examined the DSMI total score in relation to demographic and clinical variables, including sex (1 = female, 0 = male), age, occupations (1 = retirement, 2 = officers, 3 = self-employed, 4 = others), living areas (1 = urban area, 2 = others), educational status (1 = illiterate, 2 = primary school, 3 = secondary/high school, 4 = vocational/college), marital status (1 = single, 2 = married, 3 = widow/divorced), living arrangements (1 = living alone, 2 = living with relatives), duration of diabetes (1 = ≤5 years, 2 = >5 years), types of diabetes (1 = type 1, 2 = type 2), BMI, hypertention (no = 0, yes = 1), current treatment regimen (1 = OAD, 2 = insulin only, 3 = OAD + insulin), waist circumference, medical nutrition therapy (no = 0, yes = 1), sufficient physical activities (no = 0, yes = 1), dyslipidmia (no = 0, yes = 1), HbA1c, and fasting plasma glucose. Via backward selection strategy, final multiple linear regression model analysis showed that sex (β = 8.27, p = 0.031), educational status (β = 9.16, p = 0.0001), BMI (β = 1.86, p = 0.023), waist circumference, (β = -0.75, p = 0.008), medical nutrition therapy (β = 8.44, p = 0.018), and sufficient physical activity (β = 17.34, p = 0.0001) were independently predictive factors of DSMI total score, which explained 42.5% of the variance (adjusted R square = 0.425).

**Table 5:** Factors independently predictive of total DSMI score via multiple linear regression analysis.

Variable	B	95% CI of B		p
		Lower	Upper	
(Constant)	106.82	64.57	149.06	0.0001
Gender	8.27	0.79	15.75	0.031
Educational status	9.16	5.15	13.17	0.0001
BMI	1.86	0.26	3.45	0.023
Waist	-0.75	-1.29	-0.20	0.008
Medical nutrition	8.44	1.47	15.41	0.018
Sufficient physical activity	17.34	10.04	24.64	0.0001

SD, Standard Deviation; OAD, Oral Antidiabetic Drugs; CI, Confident Interval; BMI, Body Mass Index

## Discussion

Diabetes self-management can help achieve good individualized glycemic control to reduce the risk of diabetes microvascular and macrovascular complications [36, 37]. Therefore, the latest ADA guidelines for the care of diabetes include self-management behavior (SMB) as a central component in diabetes treatment [32]. In addition, any diabetes self-management interventions were based on the 5 domains of DSMI (self-integration, self-regulation, interaction with health professionals, self-monitoring blood glucose, and adherence to recommended regimen) [33].

Our study provides insights into the self-management behavior characteristics of patients with DM, the majority of whom have type 2 diabetes, who are receiving primary care in central Vietnam. Our purpose was to examine diabetes self-management and its related factors by using the V-DSMI, a validated diabetes self-management instrument. This study showed that the mean total DSM score based on the V-DSMI self-administered questionnaire was 88.4 ±22.1 and the score of 5 domains got an average of their range. In the cohort of Vietnamese patients with diabetes in this study 48.1% had good HbA1c control (HbA1c <7%) as defined by ADA 2021 criteria for classification of glycemic control. In addition, sex, educational status, BMI, waist circumference, following a medical nutrition therapy plan, and sufficient physical activity were independently predictive factors of DSMI total score.

In a comparison of DSMI scores with other regions, our outcomes are in line with the results of a study conducted in China [38] which showed that the mean DSMI score was 95.23±20.6 and mean scores for each domain of integration DM care into one’s life, self-regulations, interaction with health professionals, self-blood monitoring glucose, and adherence to the recommended regimen were 28.11, 25.22, 23.06, 10.98, and 8.75, respectively. However, our DSMI scores are lower than those found by Azar and his colleagues in Iran [33]. Their study showed that the mean total DSM score based on the DSMI self-administered questionnaire was 113.2 ± 16.89 and the mean score of each domain was greater than our result. In addition, Azar’s study found significant relationships between the total self-management score and all sociodemographic and health-related variables (p ≤ 0.001), except for a history of type 2 diabetes [33]. These differences in DSMI scores may be explained by differences in the sample sizes, healthcare systems (diabetes educational programs), healthcare settings, socio-demographic variables (educational level), and time of their study. That the current study was conducted during the COVID-19 pandemic period could potentially account for lower DSMI scores. Previous studies have reported a negative effect of COVID-19 lockdown on diabetes self-management with blood glucose levels fluctuating more during the COVID-19 lockdown being attributed to poor diet patterns, increased anxiety, and reduced physical activity levels [39–42].

Regarding other factors positively associated with DSM, the current study’ s results reveal an association of occupation and educational level with total DSMI score. This finding may be attributed to a higher educational level translating into better knowledge, attitudes and practices related to prevention and control of DM. Higher educational levels were also associated with better adherence to diabetes medications, medical nutrition therapy, and better interactions with doctors [43, 44]. Similarly, patients with higher educational levels are more likley to engage in DSM education programs and practices. In addition, retired people with DM had higher DSMI scores than others which may be attributed to having adequate time and energy to engage in DSM care regularly (a dietary plan, physical activities, or regular blood glucose check), to interact with their doctor, and/or to join diabetes classes and support groups [43]. Our study showed that participants following recommendations for medical nutrition therapy and physical activity had significantly higher DSMI scores than others. Additionally, medication adherence, medical nutrition therapy and regular physical activity are the focus of the DSM education programs which provide the knowledge and skills to help optimize glucose levels and prevent diabetes complications.

Notably, this study showed that the rate of good glycemic control was 48.1%. While this result is in line with other studies in Vietnam and other countries [45–47]. It does highlight the fact that just over 50% of persons living with diabetes in Vietnam are not considered to be in good glycemic control. Although there are now many diabetes medications available to treat people with DM, there is still a need for use of these medications to be optimized. The participants with good glycemic control (HbA1c < 7%) had higher DSMI scores than the ones with poor glycemic control (p<0.05). Clearly, engaging in DSM including adherence to DM medications helps people living with diabetes to achieve glycemic control reinforces their confidence in diabetes self-management [48]. In this study, other demographic and clinical factors were not significantly related to the total DSMI score.

The multivariate linear regression model results lead to the conclusion that DSMI total score can be predicted through sex, educational status, BMI, waist circumference, medical nutrition therapy, and sufficient physical activity. Sex plays an important role in adherence to self-management. As was the case in this study, female patients have been shown to more frequently engage in DSM, be more focused on self-care, and to search diabetes information more than males in a previous study [49]. Abdominal obesity has been shown to be a barrier to DM in self-management as those with bigger waist circumference were found to have more limitations in physical activities resulting in a obstacle to diabetes self-management [50]. Patients with DM with high BMI are generally well aware of the need to strictly follow physical activity and medical nutrition therapy regimens as well as healthy medication adherence behaviors [51].

Our study’s strengths include the application of a validated Vietnamese version of the DSMI among a sample of patients with DM in a primary care setting in Central Vietnam (Cronbach’s alpha: 0.92) and contribution of more insights into the current status of DSM and its related factors in Vietnamese DM patients. However, there are some limitations in this study. First, a cross-sectional study at a single hospital with convenience sampling may not be generalizable to the whole picture of DSM among Vietnamese patients with DM. The sample size was 78.3% of that anticipated, largely due to the COVID-19 pandemic. We were unable to use the shortened version of the DSMI which includes 20 items because it is currently unavailable in Vietnamese. The short version may be preferable for wider scale future administration, however, use of the full DSMI did allow us to obtain interesting insights into DSM in the present study. Fourth, other related variables which might affect DSM status in people with diabetes, including prior participation in DSM education known to be essential to successfully acquiring DSM skills and knowledge, psychological illness (including diabetes distress, depression, etc), medical insurance status, isolation status due to the COVID-19 pandemic, and medical treatment costs were not collected in this study. These limitations highlight the need for future research on DSM and the need for diabetes self-management education and support in adults living with DM in Vietnam.

## Conclusion

The results of this cross-sectional study of the state of DSM among patients with diabetes at a single hospital in central Vietnam demonstrates that the status of diabetes self-management may be classified as average at this time, as reflected in the mean DSMI score of 88.4 ± 22.1. Female sex, higher educational status, higher BMI and waist circumference, following a medical nutrition therapy plan and regular sufficient physical activity were independently predictive factors of DSMI total score. These findings demonstrate a need for improvement in diabetes self-management in the central region of Vietnam. There is clearly a need for further research into strategies to provide diabetes self-management education and support, particularly among those who are male, have a lower educational status and are not following medical nutrition therapy and regular physical activity regimens.

## Supporting information

S1 Data (XLSX)

## List of abbreviations

ADA: American Diabetes Association
ASMMT: Adherence and Self-Management Monitoring Tool
BMI: Body mass index
CI: Confident Interval
COVID-19: coronavirus disease 2019
DCP: Diabetes Care Profile
DHPSC: Diabetes Health Promotion Self-Care Scale
DM: diabetes mellitus
DSCAQ: Diabetes Self-Care Ability Questionnaire
DSM: Diabetes self-management
D-SMART: Diabetes Self-management Assessment Report Tool
DSMI: Diabetes Self-Management Instrument
HCPs: health care providers
MNT: medical nutrition therapy
OAD: Oral Antidiabetes Drugs
SD: Standard Deviation
SMB: self-management behavior
SMBG: self-monitoring of blood glucose
V-DSMI: Vietnamese version of the Diabetes Self-Management Instrument
